# Improved efficacy of antifungal drugs in combination with monoterpene phenols against *Candida auris*

**DOI:** 10.1038/s41598-020-58203-3

**Published:** 2020-01-24

**Authors:** Siham Shaban, Mrudula Patel, Aijaz Ahmad

**Affiliations:** 1 Clinical Microbiology and Infectious Diseases, School of Pathology, Faculty of Health Sciences, University of the Witwatersrand, Johannesburg, 2193 South Africa; 2 Infection Control, Charlotte Maxeke Johannesburg Academic Hospital, National Health Laboratory Service, Johannesburg, 2193 South Africa

**Keywords:** Fungal pathogenesis, Infection

## Abstract

Emergence of *Candida auris* has been described as a global health threat due to its ability to cause invasive infections with high mortality rate and multidrug resistance. Novel drugs and therapies are required to target this organism and its pathogenicity. Anti-virulence approach and combination therapy have been proposed as alternatives in recent years. This study evaluated the virulence factors in *C. auris*, combination antifungal activity of phenolic compounds with antifungal drugs and determined effect of the most active compound on positive pathogenicity markers of *C. auris*. Antifungal susceptibility profile of 25 clinical isolates of *C. auris* against antifungal agents as well as against phenolic compounds was obtained using CLSI guidelines. Combination of the most active phenolic compound with antifungal drugs was determined. Effect of carvacrol on the virulence factors was also studied. Carvacrol was the most active phenol with median MIC of 125 µg/ml and its combination with fluconazole, amphotericin B, nystatin and caspofungin resulted synergistic and additive effects in 68%, 64%, 96% and 28%, respectively. Combination also reduced the MIC values of the drugs. All test strains showed adherence ability to epithelial cells and 96% of strains produced proteinase. None of the strains produced hyphae and phospholipase. At low concentrations, carvacrol significantly inhibited the adherence ability and proteinase production (both p < 0.01). Carvacrol has antifungal and anti-virulence activity against *C. auris*. It also showed an enhanced antifungal activity in combination with antifungal agents. Therefore it has potential to be developed into a novel antifungal agent.

## Introduction

Emergence of *Candida auris* has been described as a global health threat. *C. auris* is typically healthcare associated pathogen. It causes highly invasive infections with high mortality rate (up to 70%) in hospitalized patients from all ages, especially those with chronic underlying disease or immunosuppression^1^. In addition, a high percentage of *C. auris* isolates have shown a resistance to one or more of the three major classes of antifungal drugs (azoles, polyenes, echinocandins) which makes the treatment difficult. Echinocandins are currently recommended as first-line of therapy for treatment of *C. auris* infections.

The pathogenicity of *Candida* spp has been attributed to virulence features, such as adherence ability, morphogenesis and the production of biofilm and hydrolytic enzymes^2^. These virulence factors are species specific and limited information is available with regards to the expression of virulence factors in *C. auris*^3,4^. Understanding of factors and activities which contribute to virulence in different *Candida* species is crucial for antifungal drug development. The concept of targeting virulence factors has been proposed as an alternative and promising antifungal strategy, since the development of new antifungal drugs is restricted by the limited number of selective drug targets in fungi^2,5^. Therefore, knowledge of these virulence factors in *C. auris*, which has been reported to be highly resistant to antifungal drugs, will assist in finding new antifungal drug targets and to develop more effective antifungal agents for improved therapeutic regimens. Moreover, the search for alternative therapies from natural sources featured by new mechanisms of action along with new strategies have become an indispensable medical priority to maintain control of this resistant fungal pathogen.

Natural plant extract, especially essential oils, are of great interest to researchers in the pharmaceutical industries due to their antimicrobial activity. Four compounds have attracted much attention, namely Eugenol, methyleugenol, carvacrol and thymol. These compounds have exhibited a potent *in vitro* fungicidal and antivirulence activity against different species of *Candida*^6,7^. Carvacrol and thymol have shown inhibitory effect against hyphae and biofilm formation in *Candida* species^8–11^. Carvacrol has been shown to be effective in reduction of the Secreted Aspartyl Proteinase (SAP) gene expression in both susceptible and resistant *C. albicans* isolates with a higher effect against the resistant isolates^12^.

Combination therapy has become an interesting area in developing new therapeutic strategies against fungal infections. They can improve the efficacy, overcome drug resistance and reduce the toxicity of antifungal drugs^13^. Several studies have shown that these phenolic compounds possess a synergistic *in vitro* antifungal activity in combination with antifungal drugs or two essential oils against different *Candida* isolates, including both susceptible and resistant strains^7,14–16^.

According to our knowledge few studies have investigated virulence factors in *C. auris* and no studies have investigated the effect of essential oils against *C. auris* and its virulence factors. This study evaluated the virulence factors in 25 isolates of *C. auris*, the combination antifungal activity of four phenolic compounds with four antifungal drugs and determined the effect of the most active phenolic compound on the positive pathogenicity markers in *C. auris*.

## Results

### Antifungal activity of antifungal agents and phenolic compounds

The MICs of 4 antifungal drugs and 4 phenolic compounds against 26 tested *Candida* strains are shown in Tables 1 and 2, respectively. MIC results depicts that *C. auris* is highly resistant to fluconazole (88%), followed by nystatin (52%). As expected control strain of *C. albicans* SC5314 was sensitive to all the four antifungal agents. MIC results of all the tested compounds showed all compounds have antifungal activity against *C. auris* strains at varying levels. From the results it is evident that carvacrol had the best MIC values (125 µg/ml) followed by thymol with MIC of 312 µg/ml for *C. auris* (Table 2). Control strain of *C. albicans* also had the lowest MIC value for carvacrol (250 µg/ml). From the results, it can also be seen that MFC values for all the four compounds are 1–2 folds higher than their respective MIC values. Based on the MFC/MIC ratios, which were lower than 4, suggested that all the four compounds have fungicidal rather than fungistatic activity^17^. Based on the MIC and MFC results, carvacrol was selected for the subsequent assays.

**Table 1:** Antifungal susceptibility of antifungal agents against *C. auris*.

*Candida* sp.	Test agents	MIC values in µg/ml (n = 3)	Interpretation No. of isolates (%)	
		Median (range)	S	R
*C. auris* (25)	Fluconazole	125 (16–500)	3 (12)	22 (88)
	Amphotericin B	0.5 (0.125–4)	20 (80)	5 (4)
	Nystatin	2 (0.5–4)	12 (48)	13 (52)
	Caspofungin	0.25 (0.125–2)	24 (96)	1 (4)
*C. albicans* (1)	Fluconazole	4 (4–8)	1 (100)	0 (0)
	Amphotericin B	0.125 (0.125–0.25)	1 (100)	0 (0)
	Nystatin	0.5 (0.25–1)	1 (100)	0 (0)
	Caspofungin	0.125 (0.125)	1 (100)	0 (0)

S: sensitive; R: resistant.

Classification based on CDC guidelines (Tentative MIC Breakpoints); Fluconazole (S < 32 µg/ml; R ≥ 32 µg/ml); Amphotericin B (S < 2 µg/ml; R ≥ 2); Nystetin (S < 2 µg/ml; R ≥ 2) Caspofungin (S < 2 µg/ml; R ≥ 2).

**Table 2:** Antifungal activity of phenolic compounds against *C. auris*.

*Candida sp*.	Test agents	MIC values in µg/ml (n = 3)	MFC values in µg/ml (n = 3)
		Median (range)	Median (range)
*C. auris* (25)	Carvacrol	125 (63–250)	250 (250–500)
	Thymol	312 (156–625)	1250 (625–1250)
	Eugenol	625 (312–1250)	2500 (625–2500)
	Methyl eugenol	1250 (625–1250)	2500 (≥2500)
*C. albicans* (1)	Carvacrol	250 (250)	500 (500)
	Thymol	625 (312–625)	1250 (625–1250)
	Eugenol	625 (625–1250)	1250 (1250–2500)
	Methyl eugenol	1250 (625–1250)	2500 (≥2500)

### Antifungal susceptibility profiling of *C. auris* isolates in combination

The results of the combination study are shown in Table 3. With the combination, the MIC values of all the antifungal agents and phenolic compound became lower than the MIC values of antifungals and phenolic compound alone. From this data, combination with carvacrol with the antifungal drugs in 1:1 ratio, indicates synergistic, additive or indifferent interactions, while no antagonistic interaction was observed. With CAR-FLU, 16% of the *C. auris* isolates showed synergistic effect of which all were resistant to fluconazole. With CAR-AMP, 28% of the *C. auris* isolates showed synergistic effect of which only 12% were resistant to AMP. With CAR-NYS, 28% of the *C. auris* isolates showed synergistic effect of which all were resistant to NYS. With CAR-CAS, only one *C. auris* isolate showed synergistic effect and it was resistant to CAS. Combination of phenolic compound and antifungal agent had no effect on the *C. albicans* control strain and all the combinations showed indifferent interactions.

**Table 3:** Antifungal activity of carvacrol in combination with antifungal agents against *C. auris*.

*Candida* sp.	Test agents	Strains (n)	MIC in µg/ml				FICI	INT No. of isolates (%)		
			MIC A	CAR A	MIC B	CAR B	Mean ± SD (range) n = 3	SYN	ADD	IND
*C. auris* (25)	CAR- FLU	Total (25)	125	125	32	63	0.83 ± 0.37 (0.29–1.51)	4 (16)	13 (52)	8 (32)
		S (3)	16	125	16	32	1.05 ± 0.30 (0.63–1.26)	0 (0)	1 (4)	2 (8)
		R (22)	125	125	32	63	0.80 ± 0.37 (0.29–1.51)	4 (16)	12 (48)	6 (24)
	CAR-AMP	Total (25)	0.5	125	0.25	4	0.67 ± 0.34 (0.28–1.50)	7 (28)	9 (36)	9 (36)
		S (20)	0.5	125	0.25	4	0.68 ± 0.30 (0.28–1.06)	4 (16)	7 (28)	9 (36)
		R (5)	2	125	0.5	8	0.61 ± 0.46 (0.31–1.50)	3 (12)	2 (8)	0 (0)
	CAR-NYS	Total (25)	2	125	0.5	4	0.49 ± 0.16 (0.28–1.03)	7 (28)	17 (68)	1 (4)
		S (12)	1	125	0.5	4	0.57 ± 0.14 (0.53–1.03)	0 (0)	11(44)	1 (4)
		R (13)	2	125	1	8	0.42 ± 0.15 (0.28–0.63)	7 (28)	6 (24)	0 (0)
	CAR-CAS	Total (25)	0.25	125	0.25	4	0.88 ± 0.25 (0.28–1.12)	1 (4)	6 (24)	18 (72)
		S (24)	0.25	125	0.25	4	0.91 ± 0.22 (0.52–1.12)	0 (0)	6 (24)	18 (72)
		R (1)	2	250	0.5	8	0.28 ± 0.00 (0.28)	1 (4)	0 (0)	0 (0)
*C. albicans* (1)	CAR- FLU	(1)	4	250	8	4	1.03 ± 0.00 (1.03)	0 (0)	0 (0)	1 (100)
	CAR-AMP	(1)	0.125	250	2	0.125	1.01 ± 0.00 (1.01)	0 (0)	0 (0)	1 (100)
	CAR-NYS	(1)	0.5	250	4	0.5	1.02 ± 0.00 (1.02)	0 (0)	0 (0)	1 (100)
	CAR- CAS	(1)	0.125	250	2	0.12	1.01 ± 0.00 (1.01)	0 (0)	0 (0)	1 (100)

FICI, fractional inhibitory concentration index; CAR, carvacrol; FLU, fluconazole; AMP, amphotericin b; CAS, caspofungin; NYS, nystatin; INT, interpretation; SYN, synergy: IND, Indifference; ADD, additive. S, sensitive; R, resistance.

MIC is the median MIC of three independent experiments. MIC A and MIC B are the median MIC of the drug alone and in combination respectively. CAR A and CAR B are the median MIC of the cravacrol alone and in combination respectively.

### Adherence and hyphae formation in *C. auris* and the effect of carvacrol on *adherence*

All the *C. auris* test strains showed varying degree of adherence ability to epithelial cells with a mean value of 141 *Candida* cell/100 epithelial cells (Table 4). *Candida albicans* showed higher adherence ability than *C. auris* (311 *Candida* cell/100 epithelial cells).

**Table 4:** Adherence of *C. auris* to buccal epithelial cells.

*Candida* sp	Strains	No. of yeast cells adherent to 100 BECs
	No. (%)	Mean ± SD (range) n = 3
*C. auris* (25)	25 (100)	141 ± 40 (67–197)
*C. albicans* (1)	1 (100)	311 ± 14 (292–324)

Microscopically, no hyphal and/or pseudohyphal cells were observed in all *C. auris* strains. In contrast, more than 90% of the *C. albicans* cells showed true hyphae within 2 hours incubation.

**Figure 1:**
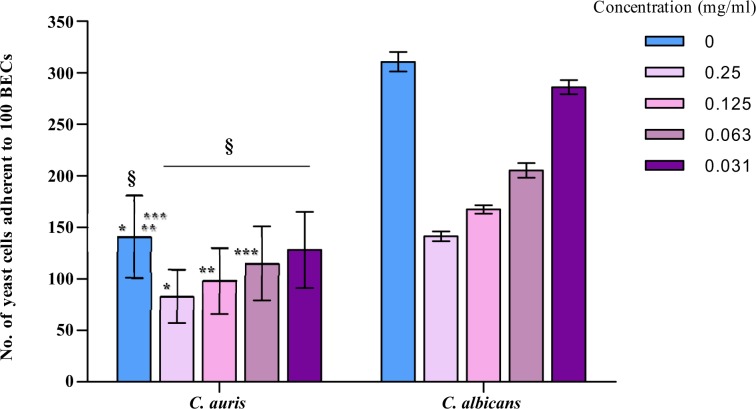
Effect of carvacrol on the adherence in *C. auris*. and *C. albicans* SC5314. ^§,*,**^p < 0.01; ***p = 0.02 - Comparison of control to various concentrations of carvacrol was done using ANOVA and Dunnett's multiple comparison test.

Having shown that carvacrol was the most active compound with the lowest MICs, *C. auris* isolates were exposed to various concentration of carvacrol to determine anti-adherence activity. Overall carvacrol reduced the adherence ability of *C. auris* significantly and the reduction was concentration dependent (Fig. 1). The significant inhibition in the adherence was at carvacrol concentration of 250 (p < 0.01), 125 (p < 0.01) and 63 µg/ml (P = 0.02). No significant inhibition was observed after exposure to 31 µg/ml carvacrol.

For *C. albicans* SC5314 adherence ability was also reduced at the tested carvacrol concentrations with the mean value (141, 167, 205 and 286) respectively, when compared to untreated cells (311) (Fig. 1). However as only one *C. albicans* SC5314 strain was used for this assay compared to *C. auris*, results are shown as mean ± SD, without further statistics.

### Proteinase and phospholipase production in *C. auris* and effect of carvacrol on proteinase production

Proteinase production was detected in 96% of the *C. auris* strains (Table 5). The quantity of proteinase was slightly lower than the *C. albicans* strain. However, 56% of the *C. auris* strains showed a very strong proteinase activity with *Pz* value of < 0.69. All *C. auris* isolates tested showed negative phospholipase activity (*Pz* = 1). In contrast, the control strain of *C. albicans* showed a very strong phospholipase activity (*Pz* = 0.66).

**Table 5:** Proteinase activity of *C. auris* isolates.

*Candida sp*.	Pz: Quantity Mean ± SD (n = 3)	Pz	
		Category	No. of strains (%)
*C. auris* (24/25) 96%	0.68 ± 0.01	++++	14 (56)
		+++	4 (16)
		++	6 (24)
		+	0
		None	1 (4)
*C. albicans* (1)	0.60 ± 0.05	++++	1 (100)

Activity level: −, Pz = 1 (no proteinase activity); +, Pz = 0.90 to 0.99 (weak proteinase activity); ++, Pz = 0.80 to 0.89 (medium proteinase activity); +++, Pz = 0.70 to 0.79 (strong proteinase activity); ++++, Pz = < 0.69 (very strong proteinase activity).

**Figure 2:**
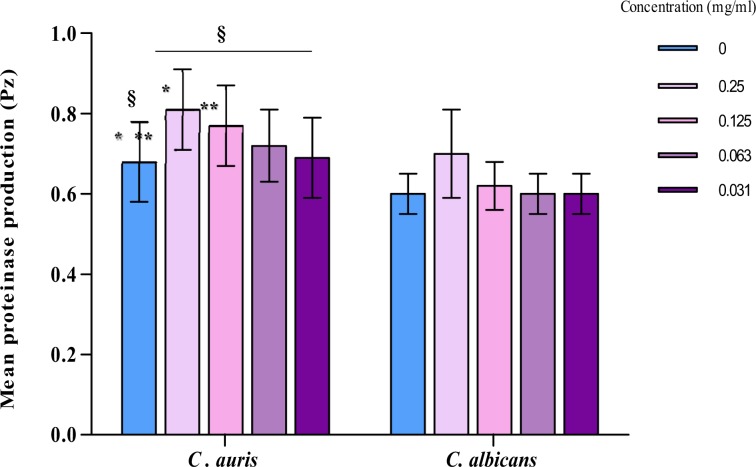
Effect of carvacrol on the proteinase production in *C. auris*. and *C. albicans* SC5314. ^§,*,**^p < 0.01 - Comparison of control to various concentrations of carvacrol was done using ANOVA and Dunnett's multiple comparison test.

Having shown that carvacrol was the most active compound with the lowest MICs against *C. auris* and proteinase production was high in this yeast. *C. auris* isolates were exposed to various concentration of carvacrol to determine to determine anti-proteinase activity. Overall carvacrol significantly reduced the proteinase production in *C. auris* with a p value of < 0.01 (Fig. 2). The significant inhibition in the proteinase production was at carvacrol concentration of 250 (p < 0.01) and 125 (p < 0.01) µg/ml. No significant inhibition was observed after exposure to 63 and 31 µg/ml of carvacrol.

Similarly, *C. albicans* SC5314 proteinase activity was reduced at carvacrol concentration of 250 (*Pz* = 0.70) and 125 µg/ml (*Pz* = 0.62) compared to the control (*Pz* = 0.60) (Fig. 2**)**. No Inhibition in the enzyme was observed at concentration of 63 (*Pz* = 0.60) and 31 µg/ml (*Pz* = 0.60) (Fig. 2**)**. All the results for *C. albicans* are represented as mean ± SD.

## Discussion

Although *C. auris* first isolated in 2009 in Japan, 10 years later it has become widespread across the globe. The uniqueness about this organism is high mortality rate of up to 72% and resistance to many common antifungal drugs. In addition, it has been found to cause nosocomial infections and significant clonal hospital outbreaks^18^. Research in antifungal drug discovery is slow and challenges with the newly emerging species of *Candida*, has made the development of new antifungal compounds and therapies a priority. This study assessed the anti-*C. auris* activity of four monoterpene phenols with and without conventional antifungal drugs. In addition, the effect of these phytochemicals on the virulence attributes of *C. auris* was also studied. The results showed that carvacrol, thymol, eugenol and methyl eugenol had antifungal activity against *C. auris* with carvacrol being the most effective monoterpene phenol. Carvacrol, being a phytochemical, has already been reported to have no cytotoxic or mutagenic effects on human cells at an MIC value of 250 µg/ml^19,20^. At higher concentrations of 1000 µg/ml, less than 20% of human blood haemolysis has been reported, which allows the *in vivo* use of carvacrol at MIC and sub-MIC values^19,21^. Besides having low MIC, at subinhibitory concentrations carvacrol also significantly inhibited the adherence to epithelial cells and reduced the proteinase production in *C. auris*. This suggests that at MIC values carvacrol can inhibit the growth of *C. auris*; while as once diluted it can render the pathogens to avirulent forms and thereby providing additional long lasting effects.

*In vitro* and *in vivo* studies of carvacrol has already been reported to possess anti-*C. albicans* activity^6,19^. In addition it also been reported to down regulate genes of aspartyl proteinase family (*SAP1, SAP2* and *SAP3*), reduce hyphae and biofilm formation in *C. albicans*^11,12,22^. *Candida* proteinases may act on the epithelial tissues and enable the fungal cells to adhere and hence penetrate in to the hosts^23^. These enzymes may also be involved in counteracting the host immune response because it is able to resist phagocytosis and intracellular killing^24^. Secretion of these enzymes is important for the pathogens as these enable them to degrade the tissue barrier and obtain nutrition at the infection site. Different proteinases are produced at different stages of infections which is associated with the morphological states of *C. albicans*, e.g., SAPs 1, 2 and 3 are expressed by the yeast phase only while as SAPs 4, 5 and 6 are expressed in the hyphal phase^25^. Production of proteinases is very well studied in *C. albicans*, however, very little is known in *C. auris*. In this study, we have shown that 96% of tested *C. auris* strains produce proteinases and these results are in line with the previous study by Larkin *et al*.^3^, where they reported 64% of the strains of *C. auris* produce proteinases. However the enzymes are not characterised and not much is known about their pattern of expression. Our results also showed that none of the strains of *C. auris* produced hyphae which suggest that SAPs 4, 5 and 6 may not be produced by this organism. Nevertheless, present study showed reduction in collective *in vitro* proteinase production with the treatment of carvacrol. Therefore this property makes carvacrol a better candidate as an antifungal agent.

This study also showed that carvacrol can inhibit the adherence ability of *C. auris*. Adhesins or surface glycoproteins are generally responsible for the adherence ability in *Candida* species^26,27^. Carvacrol may have blocked these adhesins or interfered with their synthesis and thereby reducing the adherence of *C. auris* to host cells. Although adherence and proteinase production are interlinked, in our study the effect must have been independent to one another because *Candida* proteinases generally act on the epithelial tissues and enable the fungal cells to adhere and hence penetrate. In this study, *C. auris* cells were first treated with carvacrol, washed and then exposed to the epithelial cells, which suggests that the effect of carvacrol was on the yeast cell surface. Essential oils and their compounds are known to cause damage to biological membranes, disrupting membrane permeability and inhibit respiration^16,19,28,29^.

Results in this study also revealed that ability of *C. auris* to adhere to host cells and produce proteinase is weaker than in *C. albicans*. Only 56% of the *C. auris* strains produced large quantities of proteinase, while as none of the strains produced phospholipase. These results are in contrast with the previous findings by Larkin *et al*.^3^, where some weak phospholipase activity in 37.5% of tested strains was reported^3^. In a separate study by Wang *et al*., (2018) first isolate of *C*. *auris* in China was tested for proteinase production and has been reported to exhibited high proteinase activity^4^. A case report from India has reported that *C. auris* can produce phospholipase, proteinase and have hemolysin activity^30^. These differences can be related to the geographical variations as the tested strain in our study belong to South African clade while as the strains in other studies belong to other geographical clades. Despite the differences, many studies reported weaker adherence ability of *C. auris* compared to *C. albicans* and similar results were seen in this study^3^. In addition, *C. auris* is a weaker biofilm producer and cannot produce hyphae which is crucial step in *Candida* pathogenesis^3,31^. Recent *in vivo* study has also shown that *C. auris* is weaker pathogenic *Candida* species compared to *C. albicans*^4^. And yet it has proved to be an aggressive pathogen causing outbreaks of invasive disease^32^. Perhaps its strength lies in the easy transmission, survival on inanimate objects and drug resistance. In addition, further research is required to explore other unidentified virulence traits in this *Candida* species.

Antifungal drugs are generally toxic and fungal pathogens are developing drug resistance to the less toxic drugs such as fluconazole. These problems suggest the need for new therapeutic approaches for fungal infections. Combination therapy is one of the strategy that has been explored over the years^13,33^. Antifungal agents in combination with newly identified antifungal compounds or phytochemicals can exhibit improved efficacy, broader spectrum of action, and reduced duration of therapy. In this study when the combination of commonly used antifungal agents and carvacrol was assessed, CAR-AMP and CAR-NYS showed synergistic effect in 28% of *C. auris* strains. In addition, all the test combinations showed high percentage of additive effect particularly in resistant strains. Results also show that the required dose is much less in combination compared to the test drug and carvacrol alone. Similar study was performed by Khan *et al*., (2010) who found a high rate of synergistic effect in *C. albicans* with the combination of antifungal drugs and *Ocimum sanctum* essential oil^14^. Furthermore, it would be interesting to study the effect of these combinations on the virulence properties of *C. auris*.

In conclusion, at high concentration (125 µg/ml) carvacrol has antifungal activity against *C. auris* and at subinhibitory concentrations, it inhibits the adherence ability and proteinase production. Combination of antifungal agents and carvacrol reduces the effective concentrations of both the agents with synergistic to additive effects. Therefore, carvacrol has potential to be developed into an antifungal agent.

## Materials and Methods

### *Candida cultures*, epithelial cells and ethics statement

In this study, stock cultures of *Candida auris* were used and are collected from Division of Mycology, National Institute of Communicable Diseases, Johannesburg, South Africa. All these isolates were collected with an approval by the Human Research Ethics Committee of University of the Witwatersrand (M140159) and performed according to guidelines outlined in the Helsinki Declaration. Identification was performed using Matrix-assisted laser desorption/ionization time-of-flight (MALDI-TOF) technique. In addition, one control laboratory strain *C. albicans* SC5314 was selected as positive control because its virulence factors have been well characterized. The isolates were stored in glycerol stock at −80 °C until required. Buccal epithelial cells used in adherence assay were from investigators and does not require ethical clearance.

### Antifungal susceptibility testing

Minimum inhibitory concentrations (MIC) and minimum fungicidal concentrations (MFC) were determined using broth micro dilution method following the Clinical and Laboratory Standards Institute (CLSI) guidelines M27-A3 document with slight modifications^34^. Four antifungals (fluconazole, amphotericin B, caspofungin, nystatin) and four phenolic compounds (eugenol, methyl eugenol, carvacrol and thymol) were procured from Sigma-Aldrich, St. Louis, MO, USA and respective stock solutions were prepared using 1% DMSO. A standardised inoculum of *C. auris* (10^6^ cfu/ml) was prepared by suspending *C*. *auris* colonies in 5 ml of saline. Two fold dilutions of test compounds (100 µl) were prepared in 96-well flat-bottom microtitre plates, inoculated with 100 µl of inoculum and incubated at 37 °C for 24 hours. In every set of experiment, positive control (caspofungin), negative vehicle control (1% DMSO) and culture control (media and cells only) were included. MIC endpoints were determined as the lowest concentration of drug/compound that resulted in the complete inhibition of growth or a decrease of growth by ≥ 90% relative to that of the culture control. To determine MFC, each well without visible growth was sub-cultured onto agar plates. Concentration in the first well with no growth on plate was taken as MFC. On the basis of MICs values, most active compound was selected and used for further studies. The experiment was performed in triplicate to validate the results.

### Combination study

The most active compound (carvacrol) was used in combination with fluconazole, amphotericin, nystatin and caspofungin, following a method described previously^35^. Carvacrol was combined with the antifungal drugs in a 1:1 volume ratio to the first row of microtitre plate and were serially diluted. A 100 µl of culture inoculum was added into each well and the plates were then incubated at 37 °C for 24 h, followed by MICs recording as described above. The experiment was performed in triplicate to validate the results. Based on Loewe additivity zero-interaction theory, combination interaction was calculated by determining the fractional inhibitory concentration index (FICI):

$${\rm{FICI}}={\rm{FICa}}+{\rm{FICb}}=\frac{{\rm{MICa}}\,{\rm{in}}\,{\rm{combination}}}{{\rm{MICa}}\,{\rm{tested}}\,\text{alone}\,}+\frac{{\rm{MICb}}\,{\rm{in}}\,{\rm{combination}}}{{\rm{MICb}}\,{\rm{tested}}\,{\rm{alone}}}$$

where MICa and MICb are the MICs of carvacrol and antifungal drugs, respectively. Interpretation of FICI values were synergy when ≤0.5, additive between 0.5 and 1.0, indifferent between 1.0 and 4.0 and antagonistic >4.0.

### *Candida auris* virulence factors and the effect of most active compound

All the tested *C. auris* strains were screened for the most common virulence factors including adherence, morphogenesis, phospholipase and proteinase production. *C. albicans* SC5314 was used as a control strain as all these factors are well studied in this strain. In addition, effect of the most active compound (250, 125, 63, 31 µg/ml of carvacrol) on these virulence markers was studied.

### Adherence assays

The ability of *C. auris* to adhere to the epithelial cells was determined using technique described by Patel *et al*.^36^. Buccal epithelial cells were collected from the investigator using a sterile swab. Cells were suspended, washed three times and re-suspended in 2 ml sterile distilled water. With the use of a hemocytometer, epithelial cells count was adjusted to 10^5^ cells/ml. Five millilitres of SD broth were inoculated with 10^6^ cfu of test culture and incubated at 37 °C for 24 hours. Subsequently, yeast cells were harvested by centrifugation, washed three times with distilled water and re-suspended into 2 ml of SD broth. Separately, *Candida* cells count were also standardized to 10^7^ cells/ml using a haemocytometer. Aliquots of 2 ml of yeast cells and 2 ml of oral epithelial cells were mixed and incubated at 37 °C for 2 h while shaking at 60 rpm.

To study the effect of the most active compound, *C. auris* cells were incubated for 2 hours with different concentrations of carvacrol as mentioned above, washed three times with distilled water and adjusted to 10^5^ cells/ml before mixing them with the epithelial cells. A 20 μm pore nylon filters were used to separate the epithelial cells from non-adherent yeast cells. Epithelial cells were then washed twice with distilled water and re-suspended in 1 ml of sterile distilled water. Slides were prepared and stained with the gram stain. The number of adherent yeast cells per 100 epithelial cells were measured using Leica DM 500 microscope under X1000 magnification. Each sample had a total of 3 slides and results were obtained in triplicates.

### Morphogenesis

The ability of *C. auris* to form hyphae was assessed using a protocol described by Yousuf *et al*.^37^. Briefly*, Candida* cells were sub-cultured up to late log phase at 37 °C and were then transferred into another flask containing fresh media and incubated for 48 h, for a synchronised cell population. To induce hyphal formation, 10 µl of above grown cells were transferred into 5 ml of fresh SD broth supplemented with 10% foetal bovine serum at pH 6.5. Cells were incubated at 37 °C, then aliquots of 10 µl were transferred onto a glass slide, after every 1 hour for 24 hours, a coverslip was placed and the slide was examined using Leica DM 500 microscope under X400 magnification.

### Proteinase and phospholipase production

All the *C. auris* isolates were initially screened for proteinase and phospholipase secretions using plate assay containing bovine serum albumin (BSA) and egg yolk respectively as described by Yousuf *et al*.^37^. *Candida* strains were sub-cultured in flasks containing 5 ml SD broth and incubated at 37 °C for 18 h. The cells were then centrifuged at 300 × g for 5 minutes and resuspended in fresh media. Aliquots of 2 µl were placed at equidistant points on proteinase agar plates (agar 2%, BSA 2 g, yeast nitrogen base without amino acids, ammonium sulphate 1.45 g, glucose 20 g and distilled water 1000 ml). The plates were incubated at 37 °C for 3–4 days and then examined for proteinase activity. The proteinase production was considered positive when there was a visible clear zones around the colony. Proteinase activity (*P*_*z*_ value) was assessed by measuring the ratio of the diameters of the colonies to the diameters of the clear zone around the colonies.

For the phospholipase assay, aliquots of 2 µl were placed at equidistant points on Egg Yolk plates (agar 2%, peptone 10 g, glucose 30 g, NaCl 57.3 g, CaCl_2_ 0.55 g and distilled water 900 ml, 10% egg yolk emulsion). The plates were incubated at 37 °C for 7 days. The phospholipase activity were considered positive when there was a visible precipitation zones around the colonies. Phospholipase activity (*P*_*z*_ value) was assessed by measuring the ratio of the diameters of the colonies to the diameters of the precipitation zone around the colonies.

Enzyme activity levels were classified into five classes as described by Kantarcioglu *et al*.^38^. The categories are, No proteinase activity (−: *P*_*z*_ = 1); weak proteinase activity (+: *P*_*z*_ = 0.90 to 0.99); medium proteinase activity (++: *P*_*z*_ = 0.80 to 0.89); strong proteinase activity (+++: *P*_*z*_ = 0.70 to 0.79); very strong proteinase activity (++++: *P*_*z*_ = < 0.69).

To study the effect of the active compound on secretion of both enzymes, cells from isolates showing enzyme activity were exposed to different concentrations of the most active compound for 2 hours. Cells without any treatment served as the controls and *C. albicans* SC5314 served as positive control strain. The assays were conducted on three separate occasions for each yeast isolate tested.

### Statistical analysis

All the experiments were performed in triplicate. Statistical analysis was done using the GraphPad Prism (version 5) software. A descriptive statistical analysis of the antifungal susceptibility test, the combination study and the virulence assay was performed. One-way analysis of variance (ANOVA) was used to analyse the effect of the four concentrations of carvacrol on adherence and proteinase activity of *C. auris* isolates, and then the treated groups were compared to the untreated control groups using the Dunnett’s multiple comparison test. P-value of < 0.05 was considered statistically significant.

## Data Availability

The data that support the findings of this study are available from the corresponding author upon request.
